# Supplementary material for: Modeling glioblastoma heterogeneity as a dynamic network of cell states
Source: Mol Syst Biol. 2021 Sep 16;17(9):e10105. doi: 10.15252/msb.202010105 (PMC8444284; doi:10.15252/msb.202010105)
Supplement: Supplementary file 6 — Source Data for Figure 5 [file MSB-17-e10105-s004.zip › Figure5A_sourcedata/GSEA_3071/nosigdb_stateIII.GseaPreranked.1622795155562/RICHARDS_NOWAKOWSKI_SCIENCE_2017_MGE_RG2_UPREG.html]

Details for gene set RICHARDS\_NOWAKOWSKI\_SCIENCE\_2017\_MGE\_RG2\_UPREG[GSEA]

|  || Dataset | state33071 |
| Phenotype | NoPhenotypeAvailable |
| Upregulated in class | na\_pos |
| GeneSet | RICHARDS\_NOWAKOWSKI\_SCIENCE\_2017\_MGE\_RG2\_UPREG |
| Enrichment Score (ES) | 0.3104494 |
| Normalized Enrichment Score (NES) | 4.849118 |
| Nominal p-value | 0.0 |
| FDR q-value | 0.0 |
| FWER p-Value | 0.0 |
Table: GSEA Results Summary

  

Fig 1: Enrichment plot: RICHARDS\_NOWAKOWSKI\_SCIENCE\_2017\_MGE\_RG2\_UPREG      
 Profile of the Running ES Score & Positions of GeneSet Members on the Rank Ordered List

  

| PROBE | GENE SYMBOL | GENE\_TITLE | RANK IN GENE LIST | RANK METRIC SCORE | RUNNING ES | CORE ENRICHMENT || 1 | RPL31 |  |  | 2 | 0.687 | 0.0079 | Yes |
| 2 | HSPB1 |  |  | 4 | 0.650 | 0.0156 | Yes |
| 3 | RPLP0 |  |  | 6 | 0.633 | 0.0231 | Yes |
| 4 | RPL27 |  |  | 11 | 0.587 | 0.0292 | Yes |
| 5 | RPL12 |  |  | 12 | 0.579 | 0.0363 | Yes |
| 6 | RPL6 |  |  | 16 | 0.555 | 0.0423 | Yes |
| 7 | RPL35 |  |  | 17 | 0.549 | 0.0490 | Yes |
| 8 | RPS25 |  |  | 19 | 0.527 | 0.0552 | Yes |
| 9 | RPLP1 |  |  | 25 | 0.514 | 0.0602 | Yes |
| 10 | RPL23A |  |  | 27 | 0.502 | 0.0661 | Yes |
| 11 | SNRPD2 |  |  | 29 | 0.500 | 0.0719 | Yes |
| 12 | RPS13 |  |  | 35 | 0.490 | 0.0766 | Yes |
| 13 | RPLP2 |  |  | 36 | 0.489 | 0.0826 | Yes |
| 14 | RPS6 |  |  | 41 | 0.480 | 0.0874 | Yes |
| 15 | RPL8 |  |  | 44 | 0.469 | 0.0926 | Yes |
| 16 | RPL13A |  |  | 45 | 0.467 | 0.0983 | Yes |
| 17 | SNRPC |  |  | 46 | 0.466 | 0.1041 | Yes |
| 18 | RPL5 |  |  | 51 | 0.461 | 0.1086 | Yes |
| 19 | NACA |  |  | 53 | 0.459 | 0.1140 | Yes |
| 20 | RPL32 |  |  | 55 | 0.457 | 0.1193 | Yes |
| 21 | RPL18A |  |  | 58 | 0.453 | 0.1243 | Yes |
| 22 | RPS10 |  |  | 60 | 0.452 | 0.1296 | Yes |
| 23 | RPS18 |  |  | 61 | 0.451 | 0.1351 | Yes |
| 24 | RPL36 |  |  | 62 | 0.449 | 0.1407 | Yes |
| 25 | RPL37A |  |  | 64 | 0.448 | 0.1459 | Yes |
| 26 | RPL39 |  |  | 72 | 0.442 | 0.1494 | Yes |
| 27 | RPS28 |  |  | 73 | 0.440 | 0.1548 | Yes |
| 28 | RPS3A |  |  | 82 | 0.429 | 0.1579 | Yes |
| 29 | RPS19 |  |  | 84 | 0.427 | 0.1629 | Yes |
| 30 | NPM1 |  |  | 88 | 0.421 | 0.1672 | Yes |
| 31 | RPS3 |  |  | 90 | 0.420 | 0.1721 | Yes |
| 32 | RPL10A |  |  | 94 | 0.414 | 0.1764 | Yes |
| 33 | RPS27A |  |  | 95 | 0.413 | 0.1815 | Yes |
| 34 | RPL27A |  |  | 104 | 0.408 | 0.1843 | Yes |
| 35 | RPSA |  |  | 108 | 0.405 | 0.1885 | Yes |
| 36 | SERF2 |  |  | 111 | 0.403 | 0.1929 | Yes |
| 37 | PPIA |  |  | 121 | 0.395 | 0.1953 | Yes |
| 38 | SNRPB |  |  | 122 | 0.395 | 0.2002 | Yes |
| 39 | PABPC1 |  |  | 123 | 0.394 | 0.2050 | Yes |
| 40 | RPS27 |  |  | 132 | 0.388 | 0.2076 | Yes |
| 41 | GNG5 |  |  | 139 | 0.382 | 0.2107 | Yes |
| 42 | RPL21 |  |  | 141 | 0.382 | 0.2151 | Yes |
| 43 | RPL13 |  |  | 152 | 0.372 | 0.2170 | Yes |
| 44 | GLO1 |  |  | 161 | 0.367 | 0.2193 | Yes |
| 45 | SET |  |  | 163 | 0.366 | 0.2235 | Yes |
| 46 | NME1 |  |  | 166 | 0.364 | 0.2275 | Yes |
| 47 | YBX1 |  |  | 167 | 0.364 | 0.2319 | Yes |
| 48 | RPL18 |  |  | 178 | 0.355 | 0.2336 | Yes |
| 49 | RPS15 |  |  | 180 | 0.353 | 0.2377 | Yes |
| 50 | CHCHD2 |  |  | 195 | 0.341 | 0.2381 | Yes |
| 51 | RPS14 |  |  | 196 | 0.340 | 0.2423 | Yes |
| 52 | RPL29 |  |  | 201 | 0.338 | 0.2453 | Yes |
| 53 | GSTP1 |  |  | 211 | 0.333 | 0.2470 | Yes |
| 54 | NCL |  |  | 219 | 0.328 | 0.2491 | Yes |
| 55 | RANBP1 |  |  | 221 | 0.328 | 0.2529 | Yes |
| 56 | PTMA |  |  | 222 | 0.327 | 0.2569 | Yes |
| 57 | ENO1 |  |  | 241 | 0.314 | 0.2559 | Yes |
| 58 | MZT2B |  |  | 244 | 0.313 | 0.2592 | Yes |
| 59 | RPS8 |  |  | 248 | 0.311 | 0.2622 | Yes |
| 60 | RPL23 |  |  | 250 | 0.310 | 0.2658 | Yes |
| 61 | RPL7A |  |  | 255 | 0.308 | 0.2685 | Yes |
| 62 | RPS16 |  |  | 256 | 0.308 | 0.2723 | Yes |
| 63 | RPL41 |  |  | 265 | 0.303 | 0.2738 | Yes |
| 64 | SNHG6 |  |  | 266 | 0.303 | 0.2775 | Yes |
| 65 | RPL9 |  |  | 269 | 0.302 | 0.2807 | Yes |
| 66 | HNRNPA1 |  |  | 270 | 0.302 | 0.2844 | Yes |
| 67 | TMSB4X |  |  | 273 | 0.300 | 0.2876 | Yes |
| 68 | RPL10 |  |  | 285 | 0.296 | 0.2882 | Yes |
| 69 | ANAPC11 |  |  | 286 | 0.295 | 0.2919 | Yes |
| 70 | CDC37 |  |  | 292 | 0.294 | 0.2941 | Yes |
| 71 | TIMM10 |  |  | 304 | 0.290 | 0.2947 | Yes |
| 72 | PA2G4 |  |  | 308 | 0.289 | 0.2975 | Yes |
| 73 | HMGB1 |  |  | 318 | 0.286 | 0.2986 | Yes |
| 74 | GAPDH |  |  | 320 | 0.286 | 0.3018 | Yes |
| 75 | BTF3 |  |  | 344 | 0.279 | 0.2991 | Yes |
| 76 | RPL15 |  |  | 363 | 0.271 | 0.2976 | Yes |
| 77 | COA4 |  |  | 372 | 0.269 | 0.2987 | Yes |
| 78 | CLIC1 |  |  | 373 | 0.269 | 0.3020 | Yes |
| 79 | SNRPD1 |  |  | 376 | 0.268 | 0.3048 | Yes |
| 80 | H2AFZ |  |  | 386 | 0.265 | 0.3056 | Yes |
| 81 | RAN |  |  | 388 | 0.264 | 0.3086 | Yes |
| 82 | RPL7 |  |  | 394 | 0.262 | 0.3104 | Yes |
| 83 | EIF3M |  |  | 419 | 0.253 | 0.3071 | No |
| 84 | UQCRH |  |  | 429 | 0.250 | 0.3078 | No |
| 85 | HK2 |  |  | 432 | -0.250 | 0.3103 | No |
| 86 | MNS1 |  |  | 463 | -0.252 | 0.3054 | No |
| 87 | POLE2 |  |  | 520 | -0.255 | 0.2935 | No |
| 88 | LITAF |  |  | 582 | -0.259 | 0.2803 | No |
| 89 | PTPN13 |  |  | 622 | -0.261 | 0.2731 | No |
| 90 | MCM4 |  |  | 638 | -0.261 | 0.2723 | No |
| 91 | ZFP36L2 |  |  | 671 | -0.263 | 0.2669 | No |
| 92 | PRDX2 |  |  | 708 | -0.265 | 0.2605 | No |
| 93 | ZNF473 |  |  | 781 | -0.268 | 0.2445 | No |
| 94 | CCDC181 |  |  | 797 | -0.269 | 0.2438 | No |
| 95 | MSI2 |  |  | 848 | -0.272 | 0.2337 | No |
| 96 | HES6 |  |  | 860 | -0.272 | 0.2341 | No |
| 97 | FOXN3 |  |  | 878 | -0.273 | 0.2329 | No |
| 98 | ATP1B3 |  |  | 937 | -0.276 | 0.2208 | No |
| 99 | DHFR |  |  | 956 | -0.277 | 0.2193 | No |
| 100 | LIMD1 |  |  | 962 | -0.277 | 0.2214 | No |
| 101 | HELLS |  |  | 970 | -0.278 | 0.2229 | No |
| 102 | KLF4 |  |  | 973 | -0.278 | 0.2258 | No |
| 103 | MCM3 |  |  | 1021 | -0.280 | 0.2167 | No |
| 104 | WWTR1 |  |  | 1080 | -0.284 | 0.2046 | No |
| 105 | MOB3B |  |  | 1098 | -0.284 | 0.2035 | No |
| 106 | TRIM59 |  |  | 1110 | -0.285 | 0.2041 | No |
| 107 | PCNA |  |  | 1116 | -0.286 | 0.2062 | No |
| 108 | NUSAP1 |  |  | 1262 | -0.293 | 0.1710 | No |
| 109 | DTL |  |  | 1297 | -0.295 | 0.1655 | No |
| 110 | MCM9 |  |  | 1372 | -0.299 | 0.1493 | No |
| 111 | CREB5 |  |  | 1470 | -0.304 | 0.1271 | No |
| 112 | MMS22L |  |  | 1494 | -0.306 | 0.1247 | No |
| 113 | ZHX2 |  |  | 1522 | -0.307 | 0.1212 | No |
| 114 | INPP5F |  |  | 1558 | -0.310 | 0.1156 | No |
| 115 | ZEB1 |  |  | 1569 | -0.310 | 0.1168 | No |
| 116 | ALDH1B1 |  |  | 1630 | -0.314 | 0.1045 | No |
| 117 | PLK4 |  |  | 1642 | -0.315 | 0.1054 | No |
| 118 | LIN54 |  |  | 1736 | -0.321 | 0.0845 | No |
| 119 | SCD |  |  | 1737 | -0.321 | 0.0884 | No |
| 120 | WDR76 |  |  | 1795 | -0.324 | 0.0771 | No |
| 121 | BRCA1 |  |  | 1844 | -0.327 | 0.0682 | No |
| 122 | PCDH19 |  |  | 1901 | -0.329 | 0.0573 | No |
| 123 | STARD9 |  |  | 1918 | -0.330 | 0.0570 | No |
| 124 | SALL1 |  |  | 1962 | -0.333 | 0.0496 | No |
| 125 | SMC2 |  |  | 1963 | -0.333 | 0.0537 | No |
| 126 | SLC16A1 |  |  | 2012 | -0.336 | 0.0450 | No |
| 127 | NOTCH1 |  |  | 2024 | -0.337 | 0.0461 | No |
| 128 | PCDH17 |  |  | 2043 | -0.338 | 0.0455 | No |
| 129 | LIPG |  |  | 2064 | -0.339 | 0.0443 | No |
| 130 | BRCA2 |  |  | 2213 | -0.350 | 0.0089 | No |
| 131 | MCM2 |  |  | 2303 | -0.357 | -0.0106 | No |
| 132 | FRMD5 |  |  | 2309 | -0.358 | -0.0075 | No |
| 133 | BSG |  |  | 2316 | -0.359 | -0.0047 | No |
| 134 | SPECC1 |  |  | 2368 | -0.363 | -0.0139 | No |
| 135 | ZKSCAN1 |  |  | 2437 | -0.368 | -0.0276 | No |
| 136 | PSD3 |  |  | 2464 | -0.371 | -0.0301 | No |
| 137 | PDIA6 |  |  | 2505 | -0.373 | -0.0362 | No |
| 138 | ABAT |  |  | 2521 | -0.375 | -0.0356 | No |
| 139 | IQGAP2 |  |  | 2538 | -0.376 | -0.0353 | No |
| 140 | CDC7 |  |  | 2539 | -0.376 | -0.0307 | No |
| 141 | FAM111B |  |  | 2585 | -0.381 | -0.0381 | No |
| 142 | ZRANB3 |  |  | 2594 | -0.381 | -0.0355 | No |
| 143 | METAP1D |  |  | 2646 | -0.385 | -0.0445 | No |
| 144 | PRKCE |  |  | 2692 | -0.390 | -0.0518 | No |
| 145 | EMP2 |  |  | 2695 | -0.390 | -0.0475 | No |
| 146 | CDCA7 |  |  | 2710 | -0.391 | -0.0465 | No |
| 147 | TRMT10B |  |  | 2719 | -0.392 | -0.0438 | No |
| 148 | NOTCH3 |  |  | 2759 | -0.395 | -0.0494 | No |
| 149 | FAM111A |  |  | 2761 | -0.396 | -0.0448 | No |
| 150 | SFRP1 |  |  | 2839 | -0.402 | -0.0605 | No |
| 151 | PER2 |  |  | 2864 | -0.404 | -0.0620 | No |
| 152 | SMC4 |  |  | 2872 | -0.405 | -0.0589 | No |
| 153 | LDLR |  |  | 2880 | -0.406 | -0.0558 | No |
| 154 | TLE3 |  |  | 2911 | -0.409 | -0.0588 | No |
| 155 | AASS |  |  | 2929 | -0.412 | -0.0583 | No |
| 156 | EGR1 |  |  | 2961 | -0.415 | -0.0615 | No |
| 157 | CCND2 |  |  | 3037 | -0.425 | -0.0764 | No |
| 158 | PAM |  |  | 3043 | -0.425 | -0.0725 | No |
| 159 | POLI |  |  | 3052 | -0.426 | -0.0695 | No |
| 160 | FANCD2 |  |  | 3065 | -0.427 | -0.0674 | No |
| 161 | GAB1 |  |  | 3066 | -0.428 | -0.0622 | No |
| 162 | ATAD2 |  |  | 3115 | -0.433 | -0.0697 | No |
| 163 | CCNE2 |  |  | 3138 | -0.436 | -0.0703 | No |
| 164 | HSPA5 |  |  | 3193 | -0.442 | -0.0793 | No |
| 165 | SEMA5A |  |  | 3229 | -0.447 | -0.0832 | No |
| 166 | CD63 |  |  | 3258 | -0.452 | -0.0852 | No |
| 167 | BRIP1 |  |  | 3380 | -0.475 | -0.1118 | No |
| 168 | EGFR |  |  | 3433 | -0.488 | -0.1197 | No |
| 169 | ZFP36L1 |  |  | 3487 | -0.503 | -0.1278 | No |
| 170 | SOX9 |  |  | 3560 | -0.531 | -0.1405 | No |
| 171 | CKB |  |  | 3572 | -0.536 | -0.1369 | No |
| 172 | ILDR2 |  |  | 3592 | -0.544 | -0.1353 | No |
| 173 | NES |  |  | 3596 | -0.545 | -0.1294 | No |
| 174 | DACH1 |  |  | 3607 | -0.553 | -0.1254 | No |
| 175 | SOX2 |  |  | 3613 | -0.555 | -0.1199 | No |
| 176 | FOS |  |  | 3621 | -0.561 | -0.1149 | No |
| 177 | CHD7 |  |  | 3622 | -0.561 | -0.1080 | No |
| 178 | RFTN2 |  |  | 3654 | -0.582 | -0.1092 | No |
| 179 | DLX1 |  |  | 3665 | -0.589 | -0.1047 | No |
| 180 | PDPN |  |  | 3676 | -0.597 | -0.1000 | No |
| 181 | FAT1 |  |  | 3685 | -0.604 | -0.0948 | No |
| 182 | CDK6 |  |  | 3700 | -0.614 | -0.0910 | No |
| 183 | ZBTB20 |  |  | 3702 | -0.617 | -0.0837 | No |
| 184 | SLC35F1 |  |  | 3724 | -0.635 | -0.0815 | No |
| 185 | AIF1L |  |  | 3725 | -0.635 | -0.0737 | No |
| 186 | DLX2 |  |  | 3742 | -0.662 | -0.0699 | No |
| 187 | HSP90B1 |  |  | 3757 | -0.681 | -0.0653 | No |
| 188 | RIC3 |  |  | 3785 | -0.718 | -0.0637 | No |
| 189 | SALL3 |  |  | 3787 | -0.720 | -0.0552 | No |
| 190 | MKI67 |  |  | 3788 | -0.721 | -0.0463 | No |
| 191 | TRIM9 |  |  | 3827 | -0.787 | -0.0469 | No |
| 192 | CENPF |  |  | 3842 | -0.820 | -0.0406 | No |
| 193 | ASCL1 |  |  | 3852 | -0.843 | -0.0326 | No |
| 194 | PROM1 |  |  | 3854 | -0.846 | -0.0225 | No |
| 195 | TNC |  |  | 3874 | -0.951 | -0.0160 | No |
| 196 | CYR61 |  |  | 3887 | -1.037 | -0.0065 | No |
| 197 | LMO1 |  |  | 3919 | -1.424 | 0.0027 | No |
Table: GSEA details [plain text format]

  

Fig 2: RICHARDS\_NOWAKOWSKI\_SCIENCE\_2017\_MGE\_RG2\_UPREG: Random ES distribution      
 Gene set null distribution of ES for **RICHARDS\_NOWAKOWSKI\_SCIENCE\_2017\_MGE\_RG2\_UPREG**
